# Understanding the Rising Phase of the PM_2.5_ Concentration Evolution in Large China Cities

**DOI:** 10.1038/srep46456

**Published:** 2017-04-25

**Authors:** Baolei Lv, Jun Cai, Bing Xu, Yuqi Bai

**Affiliations:** 1 Ministry of Education Key Laboratory for Earth System Modeling, Department of Earth System Science, Tsinghua University, Beijing 100084, China; 2 Joint Center for Global Change Studies (JCGCS), Beijing 100875, China

## Abstract

Long-term air quality observations are seldom analyzed from a dynamic view. This study analyzed fine particulate matter (PM_2.5_) pollution processes using long-term PM_2.5_ observations in three Chinese cities. Pollution processes were defined as linearly growing PM_2.5_ concentrations following the criteria of coefficient of determination R^2^ > 0.8 and duration time *T* ≥ 18 hrs. The linear slopes quantitatively measured pollution levels by PM_2.5_ concentrations rising rates (PMRR, μg/(m^3^·hr)). The 741, 210 and 193 pollution processes were filtered out, respectively, in Beijing (BJ), Shanghai (SH), and Guangzhou (GZ). Then the relationships between PMRR and wind speed, wind direction, 24-hr backward points, gaseous pollutants (CO, NO_2_ and SO_2_) concentrations, and regional PM_2.5_ levels were studied. Inverse relationships existed between PMRR and wind speed. The wind directions and 24-hr backward points converged in specific directions indicating long-range transport. Gaseous pollutants concentrations increased at variable rates in the three cities with growing PMRR values. PM_2.5_ levels at the upwind regions of BJ and SH increased at high PMRRs. Regional transport dominated the PM_2.5_ pollution processes of SH. In BJ, both local contributions and regional transport increased during high-PMRR pollution processes. In GZ, PM_2.5_ pollution processes were mainly caused by local emissions.

Particulate matter with diameters less than 2.5 μm (PM_2.5_) are the primary pollutants in many Chinese cities^1^. These tiny particles can readily penetrate into human lungs and bronchi^2^. Frequent severe haze episodes endanger human health and interfere with social functions. Epidemiological studies have documented adverse health effects even after short-term exposures to high PM_2.5_ concentrations^3^. Haze formation generally forms by increasing PM_2.5_ concentrations^4^. In different haze episodes, increasing PM_2.5_ concentrations may differ significantly^5^. Understanding these differences is key to determining the underlying the causes of PM_2.5_ pollution episodes^6^. These causes can include, among others, regional transport, local emissions, and unfavorable meteorological conditions.

PM_2.5_ pollution processes causes, especially in large Chinese cities, are not clearly understood. Ground observations and modeling studies have been used to analyze specific PM_2.5_ pollution episodes. In these studies, PM_2.5_ concentration, composition, and sources, along with associated meteorological conditions, are often checked to find the causes of heavy pollution episodes^7^,^8^,^9^. For example, the heavy haze pollution episodes occurring in January 2013 in North China were investigated in dozens of studies using diverse techniques. Ji *et al*. (2014) described two particulate pollution episodes in Beijing (BJ), with explosive PM_2.5_ concentration growth in one case and persistent growth in the other^5^. They concluded that the explosive episode was mainly caused by local emissions under stagnant weather conditions, while the persistent episode was largely due to normal regional transport. Other studies in BJ concluded that local emissions coupled with unfavorable meteorological factors were the main causes for prolonged PM_2.5_ pollution processes^10^. A multi-city study revealed increased secondary aerosol contributions in PM_2.5_ pollution episodes^11^. Some BJ studies concluded that PM_2.5_ pollution processes were mainly caused by regional transport from Hebei with the prevailing southern wind^10^,^12^. Meanwhile, the low boundary layer height also contributed to accumulation of local emissions^13^. Modeling studies^10^,^14^ also indicated large regional transport contributions from heavily polluted regions for PM_2.5_ pollution processes in BJ. Guo *et al*. (2014) studied the periodic cycle of PM_2.5_ pollution episodes in September 2013 in BJ to decouple multiple formation mechanisms^6^. They concluded that local secondary aerosol formation was the main cause for the pollution episodes. In all these studies, no consensus about the causes for PM_2.5_ pollution episodes was reached.

To better understand pollution conditions, an air quality monitoring network was established in China. However, long-term air quality observations, such as hourly PM_2.5_ observations, are seldom applied to pollution process analysis. The data set is often used to evaluate trends of air quality^15^, pollution levels, and spatial patterns^1^. Thus, PM_2.5_ observations are typically used to evaluate the static conditions of PM_2.5_ pollution, rather than characterizing dynamic processes. PM_2.5_ pollution is usually identified based on whether daily mean PM_2.5_ concentrations exceed the concentration limits. However, only the dynamic changes of PM_2.5_ concentrations can reveal the influences of meteorology, regional transport, or secondary reactions. The current study was performed in three large Chinese cities, BJ, Shanghai (SH) and Guangzhou (GZ). We linked a series of PM_2.5_ observations and then filter out pollution processes based on two criteria of coefficient of determinants R^2^ > 0.8 and duration time T > 18 hrs. PM_2.5_ concentration growth rates were used to indicate the strength of PM_2.5_ pollution processes. Then, PM_2.5_ pollution formation mechanisms were explored by evaluating pollution strength as it relates to meteorological variables, backward trajectories, gaseous pollutants, and regional PM_2.5_ distributions in these processes. Our results are compared with those from previous studies.

## Results

### PMRR and PM_2.5_ concentrations in three Chinese cities

**Figure 1:**
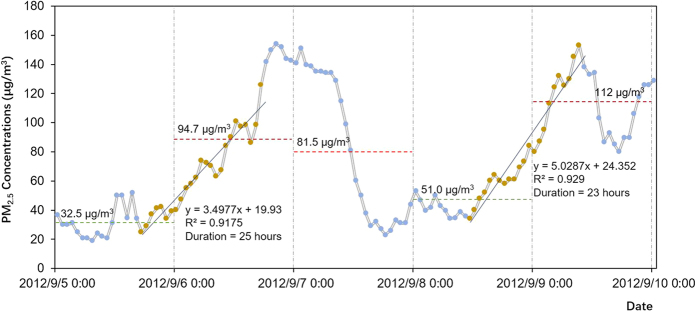
Two PM_2.5_ pollution processes in BJ and the corresponding daily mean PM_2.5_ concentrations. Brown points denote the data points within the PM_2.5_ pollution processes filtered out in this study. Horizontal dashed lines denote the daily mean PM_2.5_ levels, with the red lines referring to polluted days and the green lines referring to the attainment days measured by the static standard of 75 μg/m^3^.

**Figure 2:**
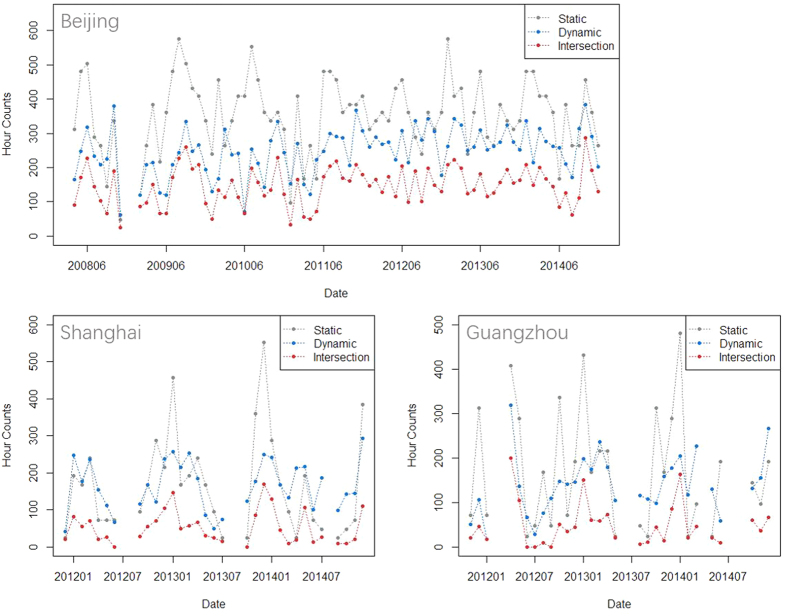
The hour counts in the static, dynamic, and intersected pollution processes in the three Chinese cities.

Examples of static and dynamic measurement of PM_2.5_ pollutions are presented in Fig. 1. Dynamic PM_2.5_ pollution processes occurred across days, while static pollution was determined by a constant standard (75 μg/m^3^ according to Chinese standard) based daily average PM_2.5_ concentration. Therefore, these measurements were independent of each other. The monthly duration hrs of PM_2.5_ pollution processes decided, respectively, by the static and dynamic measurements are presented in Fig. 2, along with the intersections of these two processes. The polluted hrs value is greatest in BJ. Generally, variability of monthly hr count in the static pollution processes is greater than that in the dynamic pollution processes. In BJ, when the monthly static pollution hrs sharply increased, the dynamic pollution hrs usually did not correspondingly increase. However, in the other two cities, the dynamic pollution hrs also increased when the static pollution hrs increased. The monthly intersected hrs exhibited similar temporal variations of monthly dynamic pollution hrs. The ratios of the intersected hrs to the dynamic polluted hrs were 0.59, 0.31 and 0.35 respectively in BJ, SH and GZ. The higher ratio in BJ indicated that the PM_2.5_ concentrations usually reached much higher values for the dynamic pollution processes in BJ than in SH and GZ.

**Figure 3:**
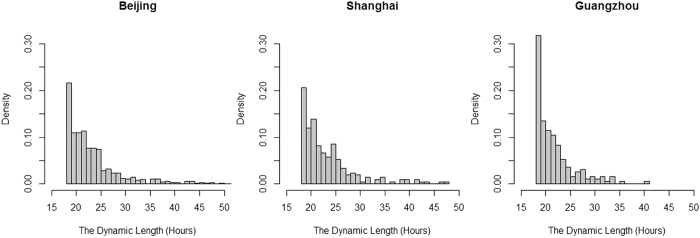
Distributions of duration time of dynamic pollution processes in three Chinese cities.

**Figure 4:**
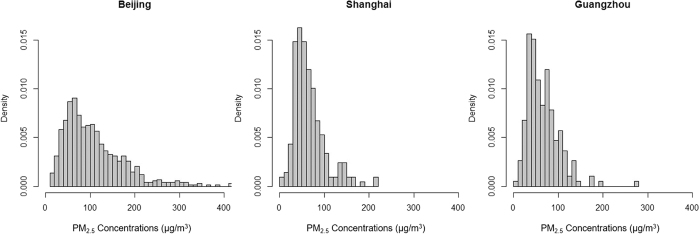
Mean PM_2.5_ levels of all the dynamic pollution processes in three Chinese cities.

The distributions of the durations of the dynamic pollution processes are similar in the three cities (Fig. 3). Generally, as the duration hrs increased, their occurrence frequencies decreased. The frequency of the marginal 18 duration hrs was significantly higher in GZ than those in BJ and SH, as shown in the Figure S1/Supplementary Information. For all the dynamic pollution processes, the average PM_2.5_ concentration distribution was different in BJ than in SH and GZ (Fig. 4). The average PM_2.5_ concentrations were more broadly distributed in BJ than in SH and GZ. The peak frequencies happened at the concentration of around the annual mean PM_2.5_ concentrations in each city, which were around 70 μg/m^3^ in BJ and 50 μg/m^3^ in SH and GZ. Meanwhile, average PM_2.5_ concentrations were concentrated below 200 μg/m^3^ in BJ and below 100 in SH and GZ. These distributions reflect the severity of PM_2.5_ pollution in BJ.

**Figure 5:**
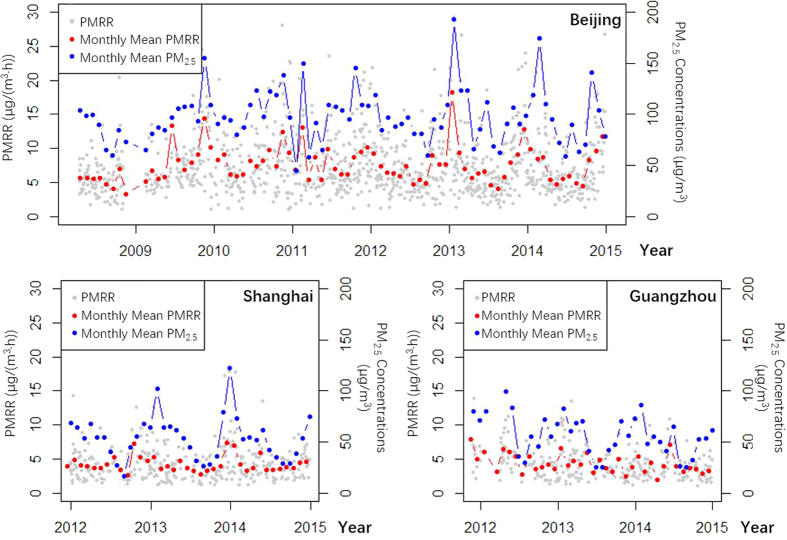
Temporal trends of PMRR, monthly mean PMRR, and monthly mean PM_2.5_ concentrations in BJ, SH, and GZ.

The long-term trends of PMRR and PM_2.5_ concentrations are both largely determined by the emission strength trends^15^. Meteorological conditions in the selected and filtered PM_2.5_ pollution processes would be relatively stable, or less variable than those in the normal situations^16^. Therefore, temporal variations of PMRR were less significant than those of PM_2.5_ concentrations (Fig. 5). The monthly mean PMRR values in the three cities decreased with statistical significance (p-value < 0.05, and from 2009 for BJ). The PM_2.5_ concentrations decreased significantly in SH and GZ, but insignificantly in BJ. The significant decrease of PM_2.5_ concentrations was also found in previous studies^17^,^18^. This indicated the consistency and wide utility of PMRR for characterizing PM_2.5_ pollution levels.

The annual periodic variations of PMRR were more obvious in BJ than those in SH and GZ (Fig. 5). In GZ, the annual periodic variations were not noticeable. In SH, variations of PM_2.5_ concentrations were much more significant than those of PMRR. By visually checking the time-series of hourly PM_2.5_ observations in SH, we found many short-time and abrupt increases of PM_2.5_ levels during winter, perhaps due to strong regional transport from North China^19^.

### Correlations with wind speed and wind direction

**Figure 6:**
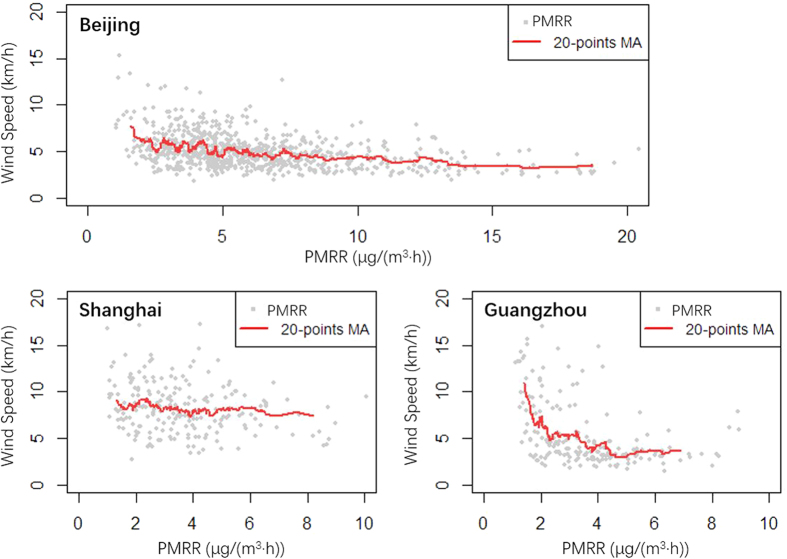
Correlations between wind speed and accumulation rates of PM_2.5_ concentrations. MA denotes the moving averages.

Wind speed and directions are key parameters determining regional transport^20^. In BJ and GZ, the PMRR exhibited linearly inverse relationships with wind speeds (Fig. 6), but in SH, the wind speeds were little changed as PMRR increased. In BJ, by calculating a 20-point moving average, the wind speed decreased in a near linear pattern from about 7 km/hr, when PMRR was around 2 μg/(m^3^·hr), to 4 km/h when PMRR was about 8 μg/(m^3^·hr). In other words, the wind speeds would decrease by about 0.5 km/h as PMRR increased by 1 μg/(m^3^·hr). Then the wind speed remained low, being less than 4 km/h, when the PMRR was larger than 8 μg/(m^3^·hr). In GZ, the two variables exhibited an inverse relationship when PMRR was less than 5 μg/(m^3^·hr). The wind speed decreased from ~10 km/hr to ~3 km/hr, while PMRR increased from ~2 μg/(m^3^·hr) to ~4 μg/(m^3^·hr). The wind speed would decrease 3.5 km/h as PMRR increased by 1 μg/(m^3^·hr). The much steeper slope indicated that the emissions or formations of PM_2.5_ pollutants were more sensitive to wind speed in GZ than in BJ. However, in SH, the relatively stable wind speed indicated that wind speed has less impact on the accumulation of PM_2.5_ pollutants than in the other two cities. In addition, the wind speed remained in a higher level in SH than in the other two cities, especially when PMRR was larger than 5 μg/(m^3^·hr).

**Figure 7:**
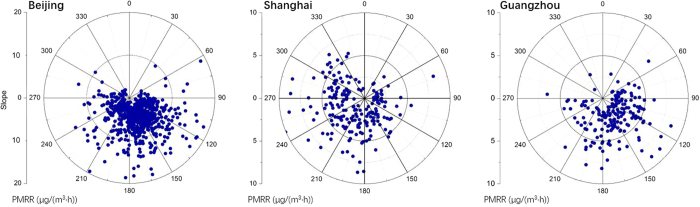
Scatter-plots of wind directions (degree, °) and accumulation rates of PM_2.5_ concentrations.

The prevailing wind directions under different levels of PMRR exhibited different features in the three cities (Fig. 7). In BJ, most of the pollution processes happened when wind directions were from southeastern, southern and southwestern directions. There are potentially two reasons for this distribution. First, the southern wind had a lower speed than winds from other directions, especially when pollution episodes occurred^5^. The low speed southern winds were favorable for the accumulation of local PM_2.5_ pollutants in BJ, which were enhanced by the mountains in the northwestern part of BJ. Second, due to the heavy pollutant emissions in the areas to the south of BJ^21^, southern winds would potentially bring large amounts of pollutants to BJ^20^,^22^. This regional transport could be an important cause for the fast-growing pollution processes. In SH, most of the pollution processes corresponded to the wind directions of western and south directions. The wind directions of high PMRR (i.e. >5 μg/(m^3^·hr)) were distributed within the scope of western directions. Regarding the low wind speeds shown in Fig. 6, we concluded that wind direction, rather than wind speed, largely determined the levels of PMRR in SH. This indicated significant contributions from regional transport to fast-growing pollution processes or large PMRR. In GZ, the pollution processes corresponded mainly to the wind directions from 60° to 270°. Considering the weak emissions surrounding PRD^21^ and low wind speeds under high PMRR (Fig. 7), high PMRR could potentially be caused by local emissions accumulations under stagnant weather conditions, rather than regional transport.

### Correlations with 24-hr backward points

**Figure 8:**
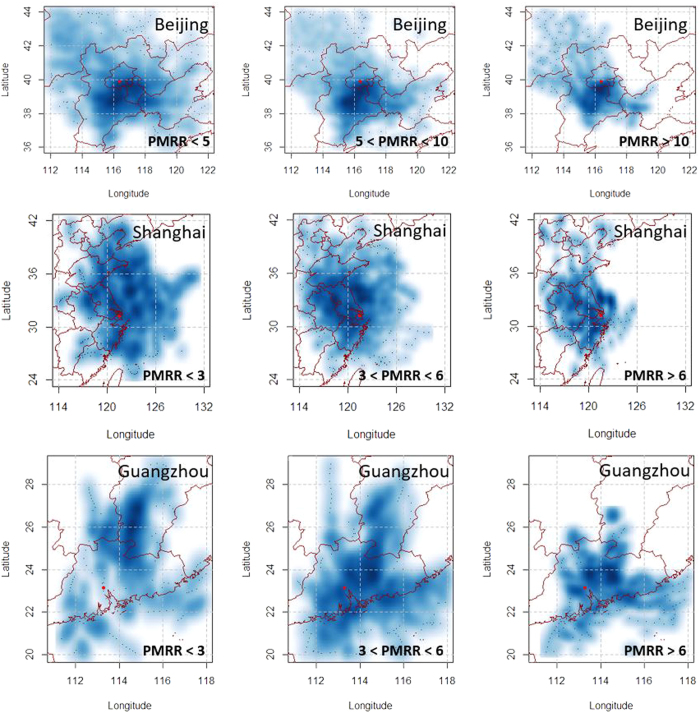
Density distribution of 24-hr backward points under different levels of PMRR (μg/(m^3^·hr)). The arriving points of air masses are the red points in the maps. The maps were drawn by R programing language^40^.

Compared to ground-level wind observations, the backward points could characterize the air mass movements on a large spatial scale. In BJ, most backward points were located to the south of BJ (Fig. 8). As PMRR increased, they became closer to the arriving points indicating lower wind speed as discussed in the previous section. When PMRR values exceeded 10 μg/(m^3^·hr), we found that the density of backward points was most intense on the boundary area between BJ and Hebei. This seems to indicate that the regional transport was weak in large PMRR scenarios, which was consistent with conclusions made by Ji *et al*.^5^ and Guo *et al*.^6^. However, severe PM_2.5_ pollutions episodes or processes in BJ usually happen as part of a regional pollution process^23^. If PM_2.5_ levels were very high in the heavily polluted area to the south of BJ, regional transport could still be significant even if weak southern winds prevailed. As PMRR increased in GZ, the backward points became closer to the arrival location. This demonstrated that regional contributions were weak, especially regarding limited emissions surrounding PRD^24^. The situation in SH was different from BJ and GZ. When PMRR values were smaller than 3 μg/(m^3^·hr), the backward points were generally randomly distributed around SH. As PMRR increased to more than 6 μg/(m^3^·hr), improved fractions of backward points were located to the northwest of SH in inland China. Meanwhile, unlike the other two cities, the distances between these backward points and arriving locations barely decreased under high levels of PMRR. Hence, rapid increases of PM_2.5_ concentrations should be closely related to regional transport.

**Figure 9:**
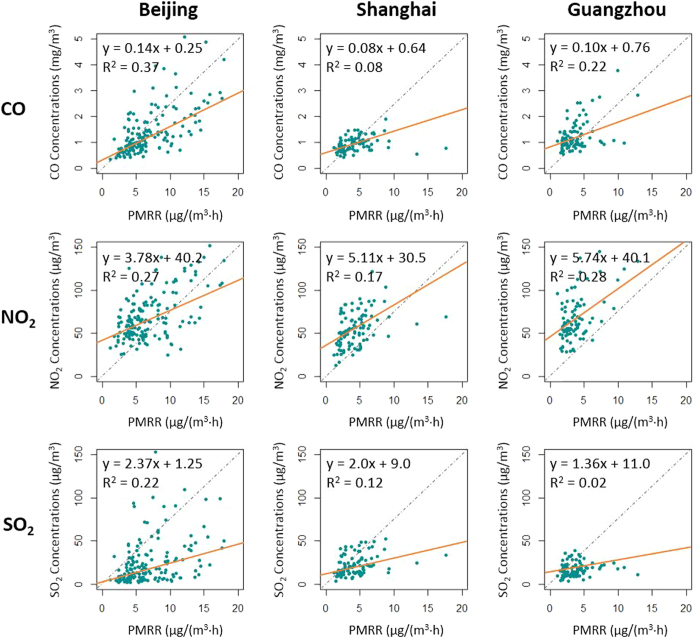
Relationships between PMRR and mean concentrations of gaseous pollutants (CO, SO_2_, and NO_2_) in the corresponding time frames in 2014. The orange lines are the regression lines generated by the Theil-Sen estimator.

In urban areas, the pollution levels of PM_2.5_ are usually linearly correlated with levels of CO^25^. Relationships between CO and PMRR were different in the three cities considering their fitting slopes and R^2^ values (Fig. 9). In the three cities, they were all in linear relationships, indicating that as PMRR values increased, the CO concentrations also increased. Unfavorable meteorological conditions such as low wind speed, which enhances PMRR, also facilitated accumulations of CO. CO is relatively chemically inert and the majority of CO is emitted within the urban area^26^. The linear relationship indicates that that more local primary emissions or secondary products of PM_2.5_ should remain in the urban area as PMRR increases. In SH, CO concentrations increased at the lowest rate as PMRR increased, indicating that contributions from locally produced PM_2.5_ increased slowly in the fast-growing PM_2.5_ pollution processes. The emission sources for NO_2_ are mainly located in urban or industrial areas^21^. The situation for NO_2_ is similar to that of CO. In the three cities, the NO_2_ concentrations increased significantly as PMRR values increased. These relationships also indicated that regional transport contributed to PM_2.5_ pollution formation in all three cities. SO_2_ was mainly emitted from areas with significant coal combustion to support heavy industry or for domestic heat^27^. The emission levels of SO_2_ were greater in North China than in YRD or PRD^27^. SO_2_ concentrations increased rapidly in BJ and SH with increased PM_2.5_ accumulation rates. Considering the strong emission levels in North China, it was clear that both BJ and SH were influenced the transport contributions from North China in the PM_2.5_ pollution processes. In GZ, SO_2_ concentrations increased slowly as PMRR enhanced, with small R^2^ values (~0.02). This indicated that there was no regional transport of SO_2_ associated with the PM_2.5_ increase in GZ.

### Regional ground-level PM_2.5_ concentrations

**Figure 10:**
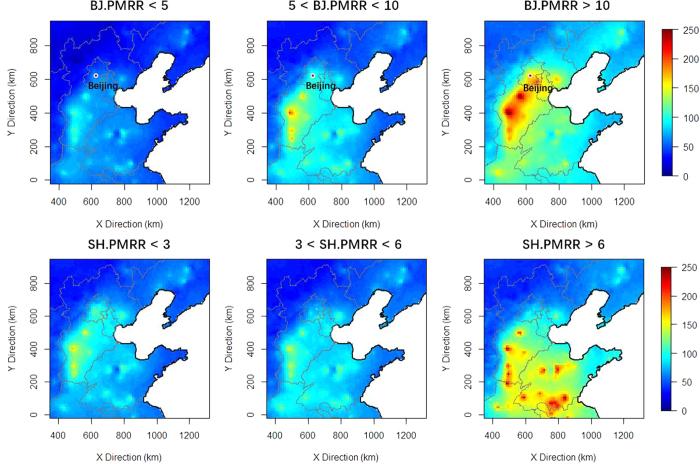
Distributions of PM_2.5_ pollution in North China under different PMRR ranges (μg/(m^3^·hr)). The maps were drawn by R programing language^40^.

Heavy PM_2.5_ pollution usually occurs on a regional scale in North China^28^. This provides potential mechanisms for regional transport to BJ in pollution episodes. The fast-growing PM_2.5_ pollution processes in BJ are closely related to the increased severity of regional PM_2.5_ pollution in the North China Plain (Fig. 10). The annual mean PM_2.5_ concentration in the Beijing-Tianjin-Hebei area in 2014 was 90 μg/m^3^. But the PM_2.5_ concentrations could reach as high as 250 μg/m^3^ when PMRR values exceeded 10 μg/(h·m^3^). When PMRR values were less than 5 μg/(h·m^3^), the maximum PM_2.5_ levels did not exceed 100 μg/m^3^. Considering the role of the prevailing southern wind in the PM_2.5_ pollution processes, it is reasonable to conclude that regional transport strength would be enhanced as the PM_2.5_ pollution process become worse (larger PM_2.5_ accumulation rates). The situation was similar in SH. As PMRR increased, the PM_2.5_ pollution levels increased in the southern part of the North China Plain, (Fig. 10). Increased PM_2.5_ pollution, along with prevailing northwestern air mass movements (Fig. 8), would facilitate the regional transport of PM_2.5_ to SH, especially when PM_2.5_ accumulation rates were high.

## Discussion

PM_2.5_ pollution processes in BJ, SH and GZ using a long-term series of hourly PM_2.5_ observations, combining with meteorological variables, 24-hr backward analysis result, and gaseous pollutant (CO, NO_2_, and SO_2_) concentrations were analyzed from a unique pollution process point of view: when PM_2.5_ concentrations increase steadily (R^2^ > 0.8) and consistently (*T* > 18 hrs). The increasing rates of PM_2.5_ concentrations (PMRR, ug/(m3 * hr)) were computed for each pollution process, and its relationship with wind speed, wind directions, 24-hr backward trajectory, concentration of CO, NO_2_ and SO_2_ were evaluated.

When local emissions play a major role in the PM_2.5_ pollution processes, it is reasonable to assume an inverse relationship between wind speed and PMRR, because strong winds would increase the dispersion of the particulate pollutants^29^. However with strong regional transport, higher wind speeds could enhance the PMRR^16^,^30^. The effects of regional transport on a specific city are usually linked to prevailing wind directions^18^ that result from fixed spatial emissions patterns. We determined the existence of regional transport in three Chinese cities. GZ was the city where PM_2.5_ pollutions was least affected by regional transport, since PMRR sharply decreased as wind speed increased. In SH, PM_2.5_ pollution was influenced by regional transport more than the other two cities, especially when PMRR was large. In BJ, regional effects were moderate compared to SH and GZ. Regional transport played a major role in SH pollution processes. In BJ, both local contributions and regional transport increased in the fast-growing pollution process. In GZ, the pollution processes were mainly caused by local emissions.

**Figure 11:**
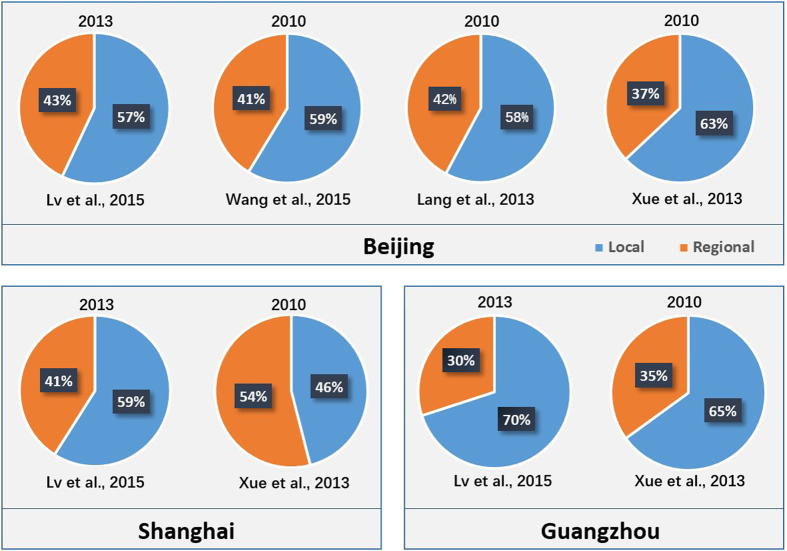
Regional and local pollution contributions based on previous studies.

Findings and conclusions from the previous studies were compared with those derived from this study, as shown in Fig. 11. In BJ, the contributions from regional transport were consistently around 40%, approximately 50% in SH, and less than 35% in GZ, using either statistical methods^18^,^22^ or chemical transport models^31^,^32^. The consistency between the results of previous studies and the conclusions in this study indicate the validity and reliability of our dynamic analysis for long-term PM_2.5_ observations. Besides the source investigations, the dynamic analysis also provided a novel approach for evaluating the temporal trend of PM_2.5_ pollution.

## Methods

### Data

#### Air pollutants observations

**Figure 12:**
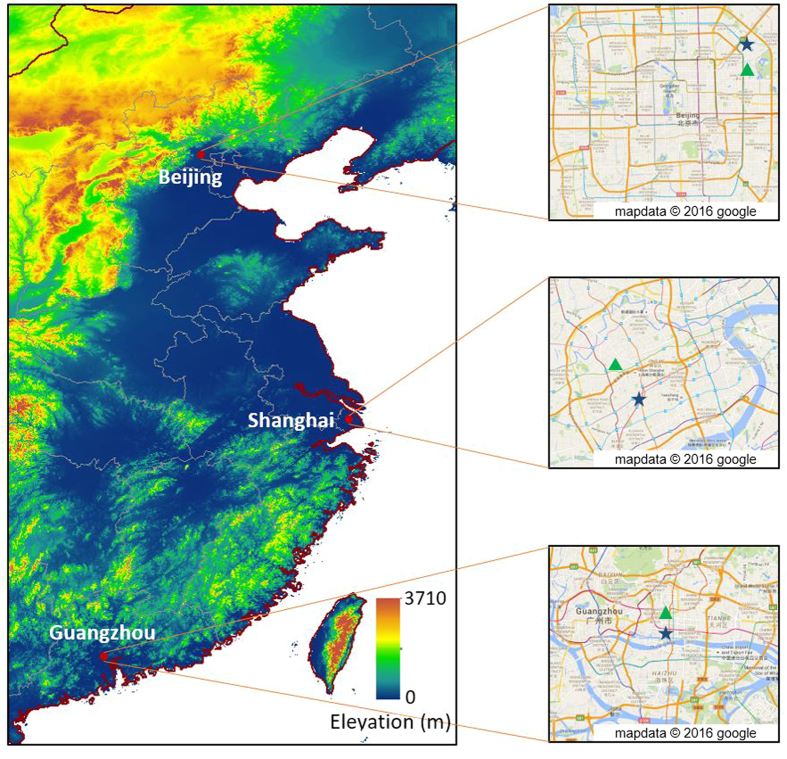
Locations of BJ, SH, and GZ, denoted as red points. The blue stars in the right panel denotes the locations of US embassy (consulates). The green triangles denote locations of the monitors for gaseous pollutants. The map in the left panel was drawn by ArcGIS (version 10.1, http://www.arcgis.com/) software and the maps in the right panel were obtained from Google maps (map.google.com).

Hourly PM_2.5_ observations in BJ, SH, and GZ are routinely monitored by the US embassy (or consulates, Fig. 12). In BJ, the data set is available from 2008 to 2014. In SH and GZ, data are available from 2011 to 2014. The Tapered Element Oscillating Microbalance (TEOM) method was used to monitor PM_2.5_ concentrations. Even though the data are not verified by embassy observers (consulates), they are in good agreement with observations by the China official monitoring network^17^.

Hourly concentrations of three gaseous pollutants, namely NO_2_, CO, and SO_2_, are monitored by the official monitoring network. The 2014 observation data were downloaded from the China National Urban Air Quality Real-time Publishing Platform (http://113.108.142.147:20035/emcpublish/), which is supported by the MEP (Ministry of Environment Protection). Their calibrations and quality controls are guaranteed by the China National Environmental Monitoring Center (CNEMC). The gaseous pollutants monitors, nearest to the PM_2.5_ monitors in the US embassy or consulates, were used, namely NongZhanGuan in BJ, JingAn in SH, and TiYuGuan in GZ (Fig. 12).

#### Meteorological Observations

We used ground meteorological observations at the airports, ZBAA, HQAP and BYAP respectively in BJ, SH and GZ. The historical hourly meteorological observations were downloaded from the National Oceanic and Atmospheric Administration (NOAA) National Climatic Data Center (NCDC, http://www.ncdc.noaa.gov/data-access). The two meteorological variables used were wind speed and wind direction. These two variables are closely related to occurrence of PM_2.5_ pollution processes^5^.

#### Ground-level PM_2.5_ concentrations

To obtain PM_2.5_ concentrations with improved spatial coverage, aerosol optical depth (AOD) retrieved from Moderate Resolution Imaging Spectroradiometer (MODIS) was always used as a proxy dataset^33^,^34^. We used the ground-level PM_2.5_ estimations in North China developed by Lv *et al*.^28^. The dataset provided us daily estimations with a complete spatial coverage in a Lambert Conic Conformal projection at a resolution of 12 km. The data set time-span was from January 10^th^ to December 31^st^, 2014.

#### 24-hr backward trajectory points

Air mass trajectories provide a convenient and effective approach to evaluate pollutant transportation pathways^22^. In this study, 24-hr air mass backward trajectory analysis was performed using the NOAA Hybrid Single Particle Lagrangian Integrated Trajectory (HYSPLIT-4) model (http://www.arl.noaa.gov/ready/open/hysplit4.html). This model is widely used to calculate dispersion and air mass trajectories^35^,^36^. The input meteorological data archive was obtained from the NCEP’s Global Data Assimilation System (GDAS) with six-hr frequency. In the study, we modeled hourly 24-hr air mass backward points arriving at BJ (39.95°N, 116.47°E), SH (31.21°N, 121.44°E), and GZ (23.12°N, 113.32°E) to investigate pollution transport pathways. The arrival height was 200 m above the ground level^18^,^37^. The 24-hr backward points in the hrs within the pollution hrs were extracted in each city.

### PM_2.5_ pollution processes identification and analysis in a dynamic way

We first defined a PM_2.5_ pollution process in a dynamic way as steady and consistent accumulations of PM_2.5_ pollutants, with the examples shown in Fig. 1. In a pollution process, PM_2.5_ concentrations were assumed to increase in a linear manner. Under this assumption, increases of PM_2.5_ concentrations, in a pollution process lasting for *T* hrs, were expressed as Eq. 1.


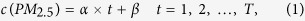


where *c (PM*_*2*.*5*_) denotes the PM_2.5_ concentration at time *t*, with *t* referring to the hr index from the beginning of a pollution process. The coefficients *α* and *β* are, respectively, the slope and intercept in the linear relationship. The slope *α* indicates the strength of a pollution process, which is referred as rising rate of PM_2.5_ concentrations (PMRR). Here, a PM_2.5_ pollution process must meet two critical criteria. First, PM_2.5_ concentrations should keep increasing for at least 18 hr, that is *T* ≥ 18. This standard is to ensure that the PM_2.5_ pollution processes are consistent under relatively stagnant meteorological conditions, rather than caused by diurnal variations^38^. Second, the R^2^ for the linear regression of a series PM_2.5_ concentrations during a specific pollution process should be ≥0.8. Good correlations support use of PMRR as an indicator for the strength of PM_2.5_ pollution processes.

Using the above two standards, we identified 741, 210 and 193 dynamic PM_2.5_ pollution processes respectively in BJ, SH and GZ from long-term observation series. The occurrence frequencies were compared between dynamic and static pollution processes. We calculated the trend of monthly mean PMRR and PM_2.5_ concentrations using a robust non-parametric Theil-Sen estimator^39^. The duration hrs, and mean PM_2.5_ concentrations of these dynamic pollution processes. were also identified and discussed. We calculated the corresponding mean values of meteorological variables, e.g., wind speed and wind direction, and concentrations of gaseous pollutants (CO, NO_2_ and SO_2_,) for these processes in 2014. The correlations between gaseous pollutants and PMRR were also calculated using the non-parametric Theil-Sen estimator in order to lower the influence of deviation values. To investigate the regional transport in PM_2.5_ pollution processes, we plotted 24-hr backward points in different PMRR value ranges. The spatial distributions of PM_2.5_ under different PMRR ranges were also discussed. For this analysis, we only used the PM_2.5_ estimations in those days when the PM_2.5_ pollution process had intersections with the time interval from 11:00 a.m. to 2:00 p.m.

## Additional Information

**How to cite this article:** Lv, B. *et al*. Understanding the Rising Phase of the PM_2.5_ Concentration Evolution in Large China Cities. *Sci. Rep.* **7**, 46456; doi: 10.1038/srep46456 (2017).

**Publisher's note:** Springer Nature remains neutral with regard to jurisdictional claims in published maps and institutional affiliations.

## Supplementary Material

Supplementary Material
