# Supplementary material for: Understanding the Rising Phase of the PM2.5 Concentration Evolution in Large China Cities
Source: Sci Rep. 2017 Apr 25;7:46456. doi: 10.1038/srep46456 (PMC5404509; doi:10.1038/srep46456)
Supplement: Supplementary Material [file srep46456-s1.doc]

# Supplementary Material of

# Understanding PM_2.5_ Pollution Process Formation by Analysis of Long-term Data

**Baolei Lv ^1,2^, Jun Cai ^1,2^, Bing Xu ^1,2,*^, Yuqi Bai ^1,2,*^**

^1^ Ministry of Education Key Laboratory for Earth System Modeling, Center for Earth System Science, Tsinghua University, Beijing 100084, China

^2^ Joint Center for Global Change Studies (JCGCS), Beijing 100875, China

^※^ Corresponding to: bingxu@tsinghua.edu.cn (Bing Xu); yuqibai@tsinghua.edu.cn (Yuqi Bai)


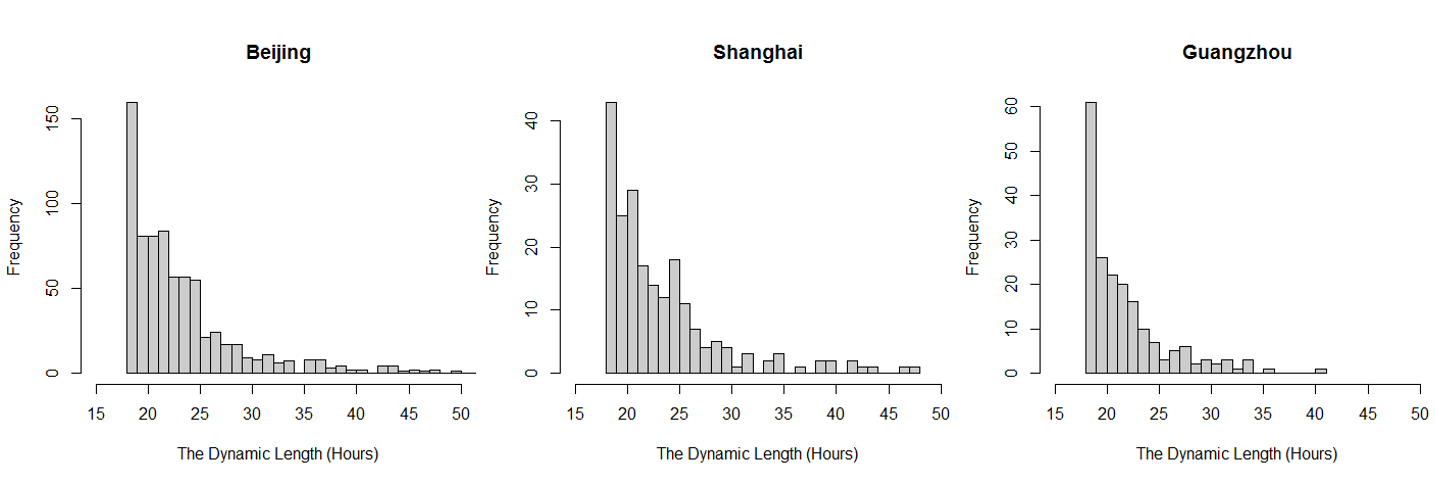


The distributions of the lengths of the dynamic pollution processes in the three cities.
